# A survey of methods for handling initial state shifts in iterative learning control

**DOI:** 10.1016/j.heliyon.2023.e22492

**Published:** 2023-11-21

**Authors:** Dongjie Chen, Tiantian Lu, Guojun Li

**Affiliations:** Basic Courses Department, Zhejiang Police College, Hangzhou, 310053, China

**Keywords:** Iterative learning control, Initial rectifying, Convergence

## Abstract

This paper introduces three types of controllers: a PID-type iterative learning controller, an adaptive iterative learning controller, and an optimal iterative learning controller, and reviews the history and research status of initial shifts rectifying algorithms. Initial state shifts have attracted research attention because they affect both the tracking performance and system stability. This study focuses on the current common initial shifts rectifying methods and analyzes the underlying mechanism in detail. To verify the effectiveness of the presented initial shifts rectifying algorithms, we simulated those using ideal first- and second-order systems. Finally, directions for the future development of iterative learning control (ILC) and some challenging topics related to initial shifts rectifying for ILC are presented. This article aims to introduce recent developments and advances in initial shifts rectifying algorithms and discuss the directions for their further exploration.

## 1 Introduction

Iterative learning control (ILC) is a data-driven control method first proposed by Uchiyama in 1978 [1], and noticed by Arimoto et al. in 1984 [2]. For repeated operations, ILC does not require a rigorous mathematical model of the control object, in general, only requires that the relevant convergence conditions are satisfied and the errors of the previous and current iterations are known [3], [4]. ILC is satisfactory owing to its complete tracking effect [5]. Over the past 40 years, ILC has made substantial advances both in theory and practice, and has been applied in the control of industrial robot, chemical batch process, factory assembly line, and multi-agent system [6], [7], [8], [9], [10], [11], [12], [13], [14].

Similar to ILC, repetitive learning control focuses on utilizing the periodicity or repetition of the target trajectory to approach the desired output by applying the input and output information from the previous cycle and improving the current control input. Repetitive learning control was first presented in 1981 [15], and its theoretical basis is the internal model principle presented by Wonham and Francis [16]. With regard to the control process, the most significant difference between the two learning control methods is that ILC is a discontinuous process, that is, the initial position must be repositioned after each iteration [3], whereas repetitive learning control is a continuous process; that is, the end position of the previous control process is the initial position of the next control process. The tracking trajectory of ILC is a curve on a closed interval, whereas that of repetitive learning control is a continuous curve with a fixed period on an infinite interval. From the control performance, ILC can realize accurate tracking over the entire interval after sufficient iterations, whereas repetitive learning control can only realize asymptotic tracking over an infinite interval [17]. Although the two control methods differ, they can suppress periodic disturbances because of their repeatability, and many applicable control ideas can be learned from each other.

Early ILC focused on improving the convergence speed and control effect. Thus far, the research goal has become more complex [18], and the scope has further expanded [19]; and the research methods [20] have become more diverse. When ILC is used, the stability and tracking effect of the system are seriously affect if the initial shifts are not zero. Owing to the positioning accuracy, measurement error and other factors, initial state error exists during repeated experiments. ILC with initial or arbitrary state shifts is a basic problem in ILC, and its study is both necessary and meaningful. Thus far, only the initial shift problem of ILC has been theoretically solved; however, these solutions have not yet been implemented. Therefore, if the system initial state error is nonzero, methods for how to completely track the reference trajectory within a specified interval has attracted the attention of scholars.

The applied controller determines the rectifying algorithm for the initial shifts rectifying of ILC. The corresponding rectifying methods vary, depending on the controller. Common iterative learning controllers are of three main types: PID-type, adaptive iterative learning, and optimal controllers. Early iterative learning controllers were primarily PID-type controllers, and their analysis methods mainly included the contraction map and 2-D methods. When the contraction map method was used for analysis, it was generally used with 2-norm, p-norm, and *λ*-norm, among which *λ*-norm was more extensive. The application of 2-D analysis methods relies on 2-D theory. Since the 1990s, adaptive iterative learning and optimal controllers have emerged in succession. The Lyapunov analysis method is generally used when adaptive ILC is applied, whereas analysis methods are diversified when optimal controllers are applied. In this work, we review the history and research status of the initial shifts rectifying methods related to controllers and analyze the mechanism in detail.

The remainder of this paper is organized as follows. Section 2 presents PID-type controllers and the corresponding rectifying algorithms, in which the tracking effects of three correction algorithms are illustrated through simulations. Adaptive iterative learning controllers and the corresponding rectifying algorithms are described in Section 3. Section 4 describes the optimal controllers and the corresponding rectifying algorithms. Finally, the conclusions are presented in Section 5.

## 2 PID-type controllers and rectifying algorithms

### 2.1 PID-type controllers

Consider the following continuous linear system described by

(1) $$\left\{ \begin{array}{c} {\overset{˙}{x}}_{k}(t)=Ax_{k}(t)+Bu_{k}(t)\\ y_{k}(t)=Cx_{k}(t) \end{array}\right.$$

where t∈[0,T]; *k* is the iteration index; and $x_{k}(t)\in R^{n},y_{k}(t)\in R^{r},u_{k}(t)\in R^{m}$ (which can be abbreviated as $x_{k},y_{k},u_{k}$, respectively) denote the state, output, and control input, respectively. A,B,C are the system parameter matrices with appropriate dimensions.

Let $e_{k}(t)=y_{d}(t)-y_{k}(t)$ represent the k-th output error, where $y_{d}(t)$ (abbreviated as $y_{d}$) and $x_{d}(t)$ (abbreviated as $x_{d}$) denote the given expected trajectory and given desired state, respectively.

Arimoto et al. [2] first presented the D-type control law as follows.

(2) $$\begin{array}{c} u_{k+1}(t)=u_{k}(t)+\Gamma {\overset{˙}{e}}_{k}(t) \end{array}$$

where Γ is the differential gain matrix. The convergence analysis is completed by applying the compression map method using the matrix norm and *λ*-norm. If there is no initial state shifts, and ‖I−CBΓ‖<1, the system (1) can achieve accurate tracking after sufficient iterations. And for nonlinear systems that satisfy the Lipschitz continuous condition, the method is still applicable. Subsequently, the following PD-type and PI-type controllers and controllers with forgetting factors have been proposed [21], [22]:

$$u_{k+1}(t)=u_{k}(t)+(\Gamma \frac{d}{dt}+\Phi )e_{k}(t)$$

$$u_{k+1}(t)=u_{k}(t)+(\Phi +\Psi \int_{0}^{t}d\tau )e_{k}(t)$$

$$u_{k+1}(t)=(1-\alpha )u_{k}(t)+\alpha u_{o}(t)+(\Phi +\Psi \int_{0}^{t}d\tau )e_{k}(t)$$

where Φ and Ψ are proportional and integral gain matrices, respectively, while *α* is a forgetting factor that satisfies 0≤α<1.

For the discrete system as follows,

(3) $$\left\{ \begin{array}{c} x_{k}(t+1)=Ax_{k}(t)+Bu_{k}(t)\\ y_{k}(t)=Cx_{k}(t) \end{array}\right.$$

among them, t=0,1,2,⋯,N; the other variables are defined similarly to the corresponding variables in system (1).

For the system (3), the following control law can be employed

$$\begin{array}{c} u_{k+1}(t)=u_{k}(t)+\Gamma e_{k}(t+1) \end{array}$$

The system can be guaranteed to converge after sufficient iterations when the initial state shifts are 0 and ‖I−CBΓ‖<1. Wang et al. [23] utilized an optimal compensation term to suppress disturbances and achieve tracking tasks when applying PID-type control laws.

After the mid-1990s, scholars began to consider ILC using a feedback term [24], [25], [26], [27], [28], [29], [30], [31]. In the 21st century, with the development of ILC, research on controlled objects has become increasingly complex, and its application has become increasingly extensive [27], [28], [29], [30], [31], [32], [33], [34]. In the past ten years, when applying the contraction map method, the research object has been more complex than the learning algorithm [29], [30], [31], [32], [33], [34].

### 2.2 Analysis methods for PID-type controllers

The convergence analysis methods for PID-type iterative learning controllers primarily include the contraction-map and 2-D methods. Owing to the simple application of the contraction map analysis method, it is omitted here. The application of 2-D analysis methods relies on 2-D theory. Consider an autonomous system described by

$$\left[\begin{array}{c} x^{h}(i+1,j)\\ x^{v}(i,j+1) \end{array}\right]=\left[\begin{array}{cc} A_{11}\; & A_{12}\\ A_{21}\; & A_{22} \end{array}\right]\left[\begin{array}{c} x^{h}(i,j)\\ x^{v}(i,j) \end{array}\right]$$

where $x^{h}$ and $x^{v}$ are state subvectors, while $A_{11}$, $A_{12}$, $A_{21}$, and $A_{22}$ are the system parameter matrices. When the system initial state is zero, Kurek et al. [35] proposed two ILC rules, i.e., with and without state feedback, established a more concise Roesser error model, and obtained a more relaxed convergence condition, namely $\rho (A_{22})<1$, where ρ(⋅) represents the spectral radius. Moreover, the convergence conditions of the Roesser error model indicate that the convergence of the ILC system is only related to the product of the system parameters and gain of the feedforward; it is unrelated to the gain of the feedback, which only accelerates or decelerates the convergence speed.

### 2.3 Initial rectifying algorithms

When the contraction map method is applied to convergence analysis, the convergence of the system is affected if there are initial state errors in the system. If the initial state errors are not corrected, the system usually can only achieve asymptotic tracking.

### 2.4 Rectifying algorithms for fixed initial shifts

For continuous systems, Porter et al. [36] presented the following control law to handle the initial state problem of ILC.

(4) $$u_{k+1}(t)=u_{k}(t)+K_{1}{\overset{˙}{e}}_{k}(t)+K_{2}{\overset{¨}{e}}_{k}(t)+K_{2}{\overset{˙}{e}}_{k}(0)\delta (t)$$

where $K_{1}$ and $K_{2}$ are gain matrices, and δ(t) is an impulse function. For a second-order system with a velocity shift, a pulse can be initially applied to the system to ensure that the initial velocity shift becomes zero, thereby avoiding this problem. However, due to the absence of pulse function in practice, this method has no practical value, but it offers ideas for solving the initial state shift problem. Heinzinger et al. [37], [38], [39] clearly indicated that when applying the D-type learning law, the tracking error is affected by the nonzero initial state shifts and their integrals. For example, in the case of system (1), when applying the control law (2), if the system has a fixed initial state value $x_{o}$, then

(5) $$\begin{array}{cc} e_{k+1}(t)=\; & y_{d}-y_{k+1}=y_{d}-y_{k}-(y_{k+1}-y_{k})\\ =\; & e_{k}(t)-\int_{0}^{t}C[A(x_{k+1}(\tau )-x_{k}(\tau ))+B\Delta u_{k}(\tau )]d\tau \\ =\; & e_{k}(t)-\int_{0}^{t}CA(x_{k+1}(\tau )-x_{k}(\tau ))d\tau -\int_{0}^{t}CB\Gamma {\overset{˙}{e}}_{k}(\tau )d\tau \\ =\; & \left(I-CB\Gamma )e_{k}(t)-\int_{0}^{t}CA(x_{k+1}(\tau )-x_{k}(\tau ))d\tau +CB\Gamma e_{k}(0\right) \end{array}$$

The above derivation shows that there is an interference term $e_{k}(0)$ that cannot be cancelled. Therefore, a fixed error exists in the first-order system. Moreover, for the high-order system, the tracking error is affected by the initial state shifts and their integrals, and the corresponding tracking error gradually increases.

Park et al. [40] applied a PID-type learning law to address initial state shifts; however complete tracking could not be achieved. Subsequently, a special nonlinear term was introduced such that the influence of the initial state shift reached zero within a specified interval [41]. This is essentially an initial rectifying. Chen et al. [42] solved the initial state shifts problem using an initial state learning scheme, and achieved accurate tracking. Considering fixed initial state shifts, Sun et al. [43], [44], [45] used an initial rectifying method for different first-order nonlinear systems to completely track the desired trajectory within a defined interval. The core elements of the control law are as follows:

(6) $$u_{k+1}(t)=u_{k}(t)+\Gamma {\overset{˙}{e}}_{k}(t)+\theta (t)\Gamma e_{k}(0)$$

The rectifying interval is [0,h], and the rectifying function θ(t) satisfies $\int_{0}^{h}\theta (\tau )d\tau =1$. If the control law (6) is substituted into (5), we obtain

$$\begin{array}{cc} \; & e_{k+1}(t)\\ =\; & y_{d}-y_{k+1}=y_{d}-y_{k}-(y_{k+1}-y_{k})\\ =\; & e_{k}(t)-\int_{0}^{t}C[A(x_{k+1}(\tau )-x_{k}(\tau ))+B\Delta u_{k}(\tau )]d\tau \\ =\; & e_{k}(t)-\int_{0}^{t}CA(x_{k+1}(\tau )-x_{k}(\tau ))d\tau -\int_{0}^{t}CB\Gamma {\overset{˙}{e}}_{k}(\tau )d\tau -\int_{0}^{t}\theta (\tau )d\tau \Gamma e_{k}(0)\\ =\; & \left(I-CB\Gamma )e_{k}(t)-\int_{0}^{t}CA(x_{k+1}(\tau )-x_{k}(\tau ))d\tau +CB\Gamma e_{k}(0)-\int_{0}^{t}\theta (\tau )d\tau \Gamma e_{k}(0\right) \end{array}$$

When t∈[h,T], there is $e_{k+1}(t)=(I-CB\Gamma )e_{k}(t)-\int_{0}^{t}CA(x_{k+1}(\tau )-x_{k}(\tau ))d\tau$. Therefore, when ‖I−CBΓ‖<1 and t∈[h,T], the initial state shifts can be corrected, and complete tracking is achieved. This is an example in which the initial correction strategy was clearly stated and adopted earlier to address the initial state errors problem. Compared with the control scheme (4) [36], the rectifying functions employed by Sun et al. [43], [44], [45] can all be practically implemented and are more feasible.

For the system (1), if A=−1, B=1 and C=1, we present control law (7):

(7) $$\begin{array}{c} u_{k+1}(t)=\left\{ \begin{array}{cc} u_{k}(t)+\Gamma {\overset{˙}{e}}_{k}(t)+\Theta (t)\Xi_{k}(0)\; & \;\;\;t\in [0,h]\\ u_{k}(t)+\Gamma {\overset{˙}{e}}_{k}(t)\; & \;\;\;t\in (h,T] \end{array}\right. \end{array}$$

where,

$$\begin{array}{c} \Theta (t)=\left\{ \begin{array}{c} \frac{2}{h}(1-\frac{t}{h})\;\;\;\;t\in [0,h]\\ 0\;\;\;\;t\in (h,T] \end{array}\right.\\ \Xi_{k}(0)=\Gamma e_{k}(0) \end{array}$$

The task interval of the system is [0,1], h=0.2 is the time point for completing the correction of the initial state shifts. The reference trajectories are $y_{d}(t)$ = $x_{d}(t)$ = sin(2πt), ${\overset{˙}{y}}_{d}(t)$ $={\overset{˙}{x}}_{d}(t)$ = 2πcos(2πt). We set the initial states such that $x_{k}(0)=0.8,{\overset{˙}{x}}_{k}(0)=0$. The simulation was iterated 16 times.

**Figure 1:**
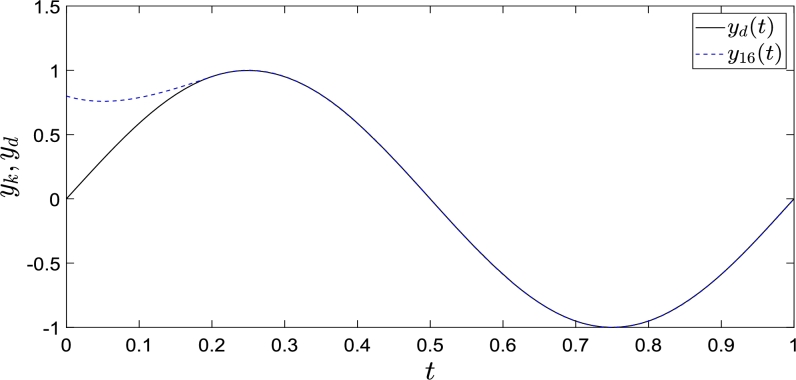
Convergence performance of *y*_*k*_ with a fixed initial shifts.

Simulations are performed to obtain Fig. 1, which shows that $y_{k}$ completely tracks $y_{d}$ in the specified interval. The dashed line represents the system outputs when the control law (7) is executed for 16 iterations, and the solid line represents the desired trajectory. Fig. 1 shows that the control law (7) completes state correction at t=0.2 and fully tracks the expected trajectory.

Subsequently, Sun et al. [46], [47] focused on nonlinear systems with high relative order under the condition that the initial state shifts were fixed and used initial rectifying methods to completely track on the system within a defined interval. Meng et al. [48], [49] respectively realized complete tracking for first- and high-order linear multi-agent systems with fixed initial state shifts, whereas Li et al. [50] achieved complete tracking for nonlinear multi-agent systems. In addition, for higher-order systems, rectifying strategies [46], [47] were used to simultaneously rectify all state shifts in a specified time, while others [49], [50] used step-by-step rectifying strategies, that is, the state shifts of the highest order were first rectified, followed by those of the next highest order, and so on, until all state shifts were rectified. When the initial state shift gradually approached a constant, the impulse compensation strategy ensured that the system achieved consistent tracking within a defined interval [51].

### 2.5 Rectifying algorithms for arbitrary initial shifts

Although Li et al. [52] attempted to use the initial rectifying strategy to solve the arbitrary initial state shifts problem under a contraction map framework, accurate tracking could not be achieved. However, this approach provides ideas for future research. In fact, for arbitrary initial state shifts, even if an initial state shifts rectifying strategy similar to (6) is adopted, at t=h, we can only obtain at most

(8) $$\begin{array}{cc} e_{k+1}(h)\; & =(I-CB\Gamma )e_{k}(h)-\int_{0}^{h}CA(x_{k+1}(\tau )-x_{k}(\tau ))d\tau \end{array}$$

As the initial state shift is arbitrary, $\int_{0}^{h}CA(x_{k+1}(\tau )-x_{k}(\tau ))d\tau \neq 0$. According to the Integral Median Theorem, $\int_{0}^{h}CA(x_{k+1}(\tau )-x_{k}(\tau ))d\tau =hCA(x_{k+1}(\varsigma )-x_{k}(\varsigma ))$, where ς∈(0,h). Therefore, for any initial state shift, even if initial state shift rectifying is adopted, achieving accurate tracking remains difficult. However, if the parameter information of the system is deterministic and can be utilized, complete tracking can still be achieved. Li et al. [53] proposed the following learning law for second-order linear systems.

(9) $$\begin{array}{c} u_{k+1}(t)=\left\{ \begin{array}{cc} u_{k}(t)+\Gamma {\overset{¨}{e}}_{k}(t)+\Theta (t)\Xi_{k}(0)-B_{R}^{-1}A[x_{2,k+1}(t)-x_{2,k}(t)]\; & \;\;\;t\in [0,h]\\ u_{k}(t)+\Gamma {\overset{¨}{e}}_{k}(t)\; & \;\;\;t\in (h,T] \end{array}\right. \end{array}$$

where,

$$\begin{array}{c} \Theta (t)=\left\{ \begin{array}{c} \frac{60}{h^{5}}(2t^{3}-3ht^{2}+h^{2}t)\;\;\;\;t\in [0,h]\\ 0\;\;\;\;t\in (h,T] \end{array}\right.\\ \Xi_{k}(0)=\Gamma e_{k}(0)+B_{R}^{-1}(x_{1,k}(0)-x_{1,k+1}(0)) \end{array}$$

We now present the following system as a simulation example.

$$\left\{ \begin{array}{c} {\overset{˙}{x}}_{1,k}(t)=x_{2,k}(t),\\ {\overset{˙}{x}}_{2,k}(t)=-x_{2,k}(t)+u(t)\\ y_{k}(t)=x_{1,k}(t) \end{array}\right.$$

The operating interval and initial shifts correction interval of the system are [0,1] and [0,0.2], respectively. The reference trajectories are $y_{1,d}(t)$ = $x_{1,d}(t)$ = cos(2πt), ${\overset{˙}{y}}_{d}(t)$ $=x_{2,d}(t)$ = −2πsin(2πt), ${\overset{¨}{y}}_{d}(t)$ = $x_{3,d}(t)=-4\pi^{2}cos(2\pi t)$. We randomize the initial states $x_{1,k}(0)=0.5rand,x_{2,k}(0)=0$, where rand denotes any value between 0 and 1.

**Figure 2:**
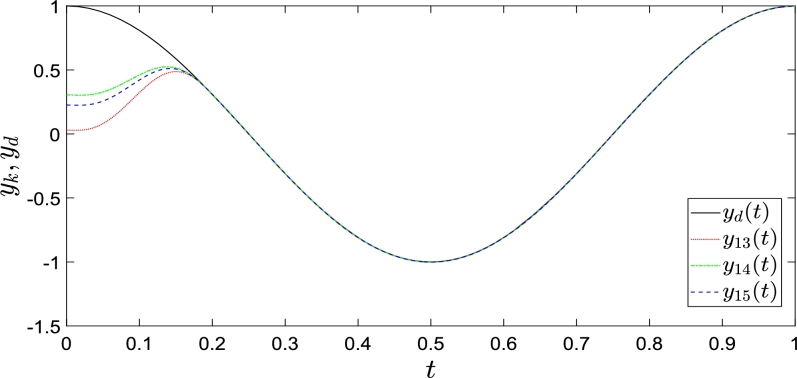
Convergence performance of *y*_1,*k*_ with arbitrary initial shifts.

**Figure 3:**
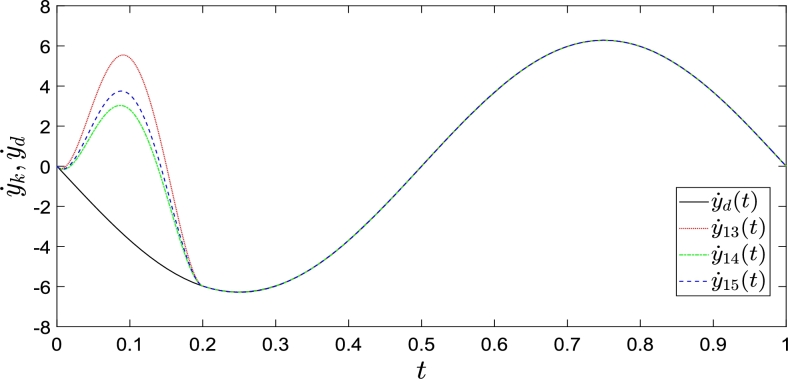
Convergence performance of *y*_2,*k*_ with arbitrary initial shifts.

The simulation results are shown in Figure 2, Figure 3, where $y_{1,k}$ and $y_{2,k}$ completely track $y_{1,d}$ and $y_{2,d}$ within a defined time, as mentioned previously. The red dotted, green dashed-dotted, and blue dashed lines represent the system outputs as control law (9) is executed 13, 14, and 15 iterations, respectively. The solid lines represent the reference outputs. Figure 2, Figure 3 show that control law (9) completes the state correction at t=0.2 and fully tracks the expected trajectory.

Similar to control law (9), Li et al. [54] proposed control laws for higher-order systems:

(10) $$\begin{array}{c} u_{k+1}(t)=u_{k}(t)+\left\{ \begin{array}{c} \Gamma_{0}e_{k}^{\left(n\right)}(t)+\sum_{i=1}^{n-1}\Theta_{i}(t)\Xi_{i,k}(h_{i,1})+\Theta_{n}(t)\Xi_{n,k}(0)+\Gamma_{1}e_{k+1}^{\left(n\right)}(t)\\ \;\;\;\;\;\;\;\;\;\;\;\;\;-{\left(CB\right)}^{-1}CA[x_{n,k+1}(t)-x_{n,k}(t)]\;\;\;t\in [0,2^{n-1}t_{p})\\ \Gamma_{0}e_{k}^{\left(n\right)}(t)+\Gamma_{1}e_{k+1}^{\left(n\right)}(t)\;\;\;\;\;\;\;\;\;\;\;\;\;\;\;\;\;\;\;\;\;\;\;\;\;\;\;\;\;\;\;\;\;\;t\in [2^{n-1}t_{p},T] \end{array}\right. \end{array}$$

where $\Gamma_{0}$ and $\Gamma_{1}$ are controller gains.

$$\begin{array}{c} \Theta_{n}(t)=\left\{ \begin{array}{c} \frac{6}{t_{p}^{3}}t(t_{p}-t)\;\;\;\;t\in [0,t_{p})\\ 0\;\;\;\;t\in [t_{p},T) \end{array}\right.\\ \Theta_{i}(t)=\left\{ \begin{array}{c} 0\;\;\;\;t\in [0,h_{i,1})\\ \overset{¯}{h_{i}}\theta_{i}\;\;\;\;t\in [h_{i,1},h_{i,2})\\ 0\;\;\;\;t\in [h_{i,2},T]) \end{array}\right.\\ h_{i,1}=2^{n-i-1}t_{p}\\ \Xi_{n,k}(0)=\Gamma_{0}e_{k}^{\left(n-1\right)}(0)+\Gamma_{1}e_{k+1}^{\left(n-1\right)}(0)\\ \;\;\;\;\;\;\;\;\;\;\;\;+B^{-1}(x_{n,k}(0)-x_{n,k+1}(0))\\ \Xi_{i,k}(h_{i,1})=\Gamma_{0}e_{k}^{\left(i-1\right)}(h_{i,1})+\Gamma_{1}e_{k+1}^{\left(i-1\right)}(h_{i,1})\\ \;\;\;\;\;\;\;\;\;\;\;\;+B^{-1}(x_{i,k}(h_{i,1})-x_{i,k+1}(h_{i,1}))\neq \end{array}$$

where

$$\begin{array}{c} h_{i,2}=2h_{i,1}\\ \overset{¯}{h_{i}}=\frac{2}{{\left(h_{i,1}\right)}^{4(N-i)+2}}\frac{[2(N-i)+1]!}{{\left((N-i)!\right)}^{2}}\\ \theta_{i}={\left(t^{N-i}{\left[t-h_{i,1}\right]}^{2(N-i)+1}{\left[h_{i,2}-t\right]}^{N-i}\right)}^{\left(n-i\right)} \end{array}$$

$t_{p}$ and N>n are a given positive real number and a positive integer, respectively. $\theta_{i}$ is the (n−i)th derivative of $t^{N-i}{\left[t-h_{i,1}\right]}^{2(N-i)+1}{\left[h_{i,2}-t\right]}^{N-i}$. For any function $\Theta_{i}(t)$, when $t_{0}\in [h_{i,2},T]$, we can obtain:

$$I_{q}=\underset{q}{\underset{︸}{\int_{0}^{t_{0}}\cdots \int_{0}^{t_{q-1}}}}\Theta_{i}(t_{q})dt_{q}\cdots dt_{1}=\underset{q}{\underset{︸}{\int_{h_{i,1}}^{h_{i,2}}\cdots \int_{h_{i,1}}^{t_{q-1}}}}\Theta_{i}(t_{q})dt_{q}\cdots dt_{1}=\left\{ \begin{array}{c} 1\;\;\;\;q=n-i+1\\ 0\;\;\;\;0<q<n-i+1 \end{array}\right.$$

The control law (10) rectifies the state shifts in a sequential order; $x_{n,k}$ and $x_{1,k}$ have the highest and lowest priorities, respectively. The state error of $x_{n,k}$ is rectified first, followed by the errors of $x_{n-1,k},x_{n-2,k},\cdots ,x_{2,k},x_{1,k}$, respectively.

In fact, irrespective of whether $B_{R}^{-1}A[x_{2,k+1}(t)-x_{2,k}(t)]$ in the control law (9), or ${\left(CB\right)}^{-1}CA$ $\left[x_{n,k+1}(t)-x_{n,k}(t)\right]$ in the control law (10) are considered, they eliminate the effect of $\int_{0}^{h}CA(x_{k+1}(\tau )-x_{k}(\tau ))d\tau \neq 0$ in (8). However, when the parameter information of the system is unknown, complete tracking under the application of the control laws (9) and (10) cannot be realized. Therefore, in a sense, control laws are only of theoretical significance and are not practical. Using the averaging operator, the algorithm can correct the initial state shift [55], which provides a reference for rectifying the initial state shifts. Meng et al. [56] achieved accurate tracking within a defined interval using the average operator and initial correction algorithm and solved the initial value problem of ILC to a certain extent.

For discrete systems, complete tracking is difficult to achieve if there is an initial state shift, because the discrete system cannot perform initial shift rectifying through integration. Saab [57] showed that the tracking error is globally bounded in the presence of state disturbances, measurement noise, and a re-initialization shift. Hillenbrand et al. [58] indicated that the system would not achieve ideal tracking if the initial state shift of each iteration was random. For the high relative degree nonlinear discrete-time systems, Sun et al. [59] presented sufficient conditions for convergence and noted that the limit of the output error was proportional to the initial state shift. For fixed values of the initial state shift, the initial rectifying method [60], [61] enables the system to completely track the desired trajectory over a predetermined interval; however, this method was only suitable for a fixed initial state shift. For arbitrary initial state shifts, this method only reduced the tracking error but could not eliminate the error.

When the contraction map method is used for convergence analysis, the feed-forward or causal learning law is generally used; that is, the learning law for a continuous system should contain ${\overset{˙}{e}}_{k}(t)$ or higher order derivatives, whereas that for discrete systems should contain $e_{k}(t+1)$. In this case, if the initial state shift is zero, the tracking error gradually approaches zero after sufficient iterations. If the initial state shift is fixed, the aforementioned rectifying methods are applied, and the system can still realize ideal tracking after sufficient iterations within the defined interval. Currently, for the random initial value problem of the system, the adoption of an initial rectifying strategy under the contraction map framework to ensure that the system achieves complete tracking within a specified interval remains an open topic.

## 3 Adaptive iterative learning controllers and rectifying algorithms

### 3.1 Adaptive iterative learning controllers

Adaptation is the ability to modify its own characteristics to adjust to the dynamic changes in a controlled object. Its basic idea can be summarized as follows: when the system parameters or the characteristics of the controlled object are unknown, the control law is designed such that the system can automatically adjust the control law, track the controlled object according to the actual situation, and achieve the desired results.

A robust adaptive iterative learning controller (AILC) presented in [62] can ensure high-precision tracking for the spacecraft with parameter uncertainties and external disturbances. Bu et al. [63] focused on designing an AILC using projection recognition algorithms along the iterative axis. This control algorithm ensured that the parameter estimation converged within a finite interval and the tracking error converged asymptotically point-by-point. An AILC was developed for discrete nonlinear systems with arbitrary initial conditions [64], unknown time-varying input gains and parameters, external disturbances and so on. Li et al. [65] proposed a constrained spatial AILC to realize displacement-speed trajectory tracking for an automatic train control system with speed constraints and unknown uncertainties. The AILC [66] ensured that the system achieved complete tracking by rectifying initial shifts. For parameterizable nonlinear systems, Xu et al. [67] combined variable structure control and learning control and presented a new nonlinear control scheme, namely, robust AILC, which can also be applied to nonlinear systems with dead zones [68]. In fact, applying a model-free AILC could also achieve consensus tracking for nonlinear multi-agent systems [69]. These studies present a reference for the wider application of adaptive methods to ILC.

### 3.2 Lyapunov-like analysis method

Stability theory, which was proposed by the Russian mathematician Lyapunov in 1892, is now widely used in analytical mechanics and automatic control systems. Since the 1990s, convergence analysis techniques combining the Lyapunov stability theory and adaptive methods have been successively applied to ILC and repetitive control, resulting in some progress. Earlier, Sadegh et al. [70] applied the adaptive control method to the controller design for repetitive control and introduced the following Lyapunov-like function (11) for convergence analysis. The composite energy function [71] was not significantly different; however, its convergence analysis method has been adopted in the subsequent studies. Xu et al. [72] provided strategies that could learn from different tracking tasks and ensure complete tracking.

(11) $$V_{k}(t,e_{k}(t))=\frac{1}{2}e_{k}^{\text{T}}(t)e_{k}(t)+\frac{1}{2}\int_{0}^{t}{\overset{˜}{\omega }}_{k}^{\text{T}}(\tau )K_{L}^{-1}{\overset{˜}{\omega }}_{k}(\tau )\text{d}\tau$$

Here, ${\overset{˜}{\omega }}_{k}(t)$ is the parameter estimation error, and $K_{L}$ is a positive definite learning gain matrix, which is limited by the saturation function during learning.

Chen et al. [73] applied a backstepping method to achieve global stability of an adaptive neural network system. For time-delay nonlinear systems with time-varying parameters, Li et al. [74] employed adaptive ILC to achieve system stability.

### 3.3 Initial rectifying algorithms

Many satisfactory results have been obtained by utilizing the Lyapunov stability analysis method to study the initial state shifts. Although Park et al. [75] did not aim at repeating systems, their proposed terminal sliding mode control scheme for second-order nonlinear uncertain systems solved the tracking problem with nonzero initial state shifts. The use of the sliding hyperplane ensured the complete convergence of the system. The idea of the initial correction can be seen in the control scheme [75]. If the uncertainty of a nonlinear system is bounded, asymptotic tracking can be achieved under arbitrary initial conditions by designing state-feedback ILC [76]. Xu et al. [77] summarized the intrinsic relations between five different initial conditions and the corresponding learning convergence properties. Sun et al. [78] introduced the convergence of function sequences within a finite time interval and applied it to convergence analysis for ILC, particularly for the convergence analysis of nonzero initial state shift systems.

#### 3.3.1 Asymptotic tracking initial rectifying algorithms

Chien et al. [79], [80] introduced a boundary layer that converged monotonically to zero along the time axis. The following design ideas were adopted: We define

(12) $$s^{j}(t)=c_{1}e_{1}^{j}(t)+c_{2}e_{2}^{j}(t)+\cdots +c_{n-1}e_{n-1}^{j}(t)+c_{n}e_{n}^{j}(t)$$

and

$$\begin{array}{c} s_{\phi }^{j}(t)=s^{j}(t)-\phi^{j}\text{sat}(\frac{s^{j}(t)}{\phi^{j}(t)})\\ \phi^{j}(t)=\varepsilon^{j}exp(-kt) \end{array}$$

Here, *j* denotes the number of iterations; *exp* denotes the exponential function; and **sat** is the saturation function, which is defined as follows:

$$\text{sat}(\frac{s^{j}(t)}{\phi^{j}(t)})=\left\{ \begin{array}{cc} 1,\;\;\; & \;\;if\;\;s^{j}(t)>\phi^{j}(t)\\ \frac{s^{j}(t)}{\phi^{j}(t)},\;\;\; & \;\;if\;\;|s^{j}(t)|\leq \phi^{j}(t)\\ -1,\;\;\; & \;\;if\;\;s^{j}(t)<-\phi^{j}(t) \end{array}\right.$$

Then, the control law is designed to make $s_{\phi }^{j}(t)$ asymptotically approach 0 under the limit of the boundary layer $\phi^{j}(t)$. It should be noted that $\phi^{j}(t)$ constrains $s_{\phi }^{j}(t)$, not $e_{1}^{j}(t)$, $e_{2}^{j}(t)$, ⋯, $e_{n}^{j}(t)$, i.e., $e_{1}^{j}(t)$, $e_{2}^{j}(t)$, ⋯, $e_{n}^{j}(t)$ may cross the limit of the boundary layer $\phi^{j}(t)$. For example,

(13) $$s^{j}(t)=c_{1}e_{1}^{j}(t)+c_{2}e_{2}^{j}(t)+e_{3}^{j}(t)$$

If $s^{j}(t)\equiv 0$, (13) obviously represents a second-order homogeneous differential equation, whose characteristic equation of the form

(14) $$0=c_{1}+c_{2}r+r^{2}$$

Because the coefficients $c_{1}$ and $c_{2}$ satisfy Hurwitz's theorem, it can be ensured that the eigenvalues of the characteristic equation (14) $r_{1}<0$, $r_{2}<0$. Correspondingly, the tracking error $e_{1}^{j}(t)=a_{1}exp(r_{1}t)+a_{2}exp(r_{2}t)$, where $a_{1}$ and $a_{2}$ are related to the initial values. Therefore, setting reasonable parameters $c_{1}$ and $c_{2}$ can yield $a_{1}=0$, $a_{2}=0$, and $e_{1}^{j}(t)=0$, so that the system output completely tracks the reference over the entire interval. However, when $s^{j}(t)\neq 0$, differential equation (13) is an inhomogeneous linear differential equation. Thus $e_{1}^{j}(t)$ is affected by $exp(r_{1}t)$, $exp(r_{2}t)$, and $s^{j}(t)$. At this point, it is difficult to achieve that for any parameter $c_{1}$, $c_{2}$, there exist $a_{1}=0$, $a_{2}=0$, and $e_{1}^{j}(t)=0$. However, the asymptotic convergence of $e_{1}^{j}(t)$ can still be realized by selecting the appropriate parameters.

Lv et al. [81] designed the following sliding mode error function:

(15) $$\sigma_{k}(t)=c_{n}e_{k}^{\left(n\right)}(t)+\cdots +c_{i}e_{k}^{\left(i\right)}(t)+\cdots +c_{0}e_{k}(t)$$

When the input gain of the system is known, initial rectifying can ensure that the value of $\sigma_{k}(t)$ transitions from $\sigma_{k}(0)$ to 0 within the specified interval. The above analysis shows that although the value of $\sigma_{k}(t)$ is equal to 0 in the specified interval, it does not guarantee that $e_{k}^{\left(i\right)}(t)=0,i=0,1,\cdots ,n$ (the meaning of the subsequent *i* is the same as here) because $\sigma_{k}(0)\neq 0$. Based on the form of the differential equation solution, this method can only ensure that $e_{k}^{\left(i\right)}(t)$ asymptotically tends to 0. Therefore, the method can only achieve asymptotic tracking within a specified interval as the number of iterations increases.

Similarly, for uncertain systems with variable system parameters along the time axis and iterative axis, when the initial state was fixed, Yin et al. [82] achieved asymptotic convergence of the system by using a high-order internal model control method.

#### 3.3.2 Complete tracking initial rectifying algorithms

Sun et al. [83] introduced the attractor for first-order systems, which is defined as follows: If $\chi (t)=0,t>t_{1}$, where $t_{1}<\infty$, then χ(t) is called a finite-time attractor. We have made appropriate modifications and extensions based on this definition.

Definition 1 If

(16) $$\overset{˙}{\chi }(t)+a\chi (t)+r(t)=0$$

where, χ(t),r(t)∈R, *a* are constants, and if $\chi (t)=0,\overset{˙}{\chi }(t)=0$ when $t>t_{1}$, then r(t) is called the initial rectifying function of the first-order attractor.

In equation (16), we choose r(t) as follows

$$\begin{array}{ccc} r(t)\; & =\; & \zeta (t)\exp(-a(t-t_{0})+\chi (t_{0})), \end{array}$$

where ζ(t) is an undetermined function. According to the equation (16), there is

$$\begin{array}{cc} \chi (t)=\; & (\chi (t_{0})-\exp(\chi (t_{0}))\int_{t_{0}}^{t}\zeta (\tau )\text{d}\tau )\cdot \exp(-a(t-t_{0})). \end{array}$$

Differentiating χ(t), we obtain

$$\begin{array}{ccc} \overset{˙}{\chi }(t)\; & =\; & -(\chi (t_{0})-\exp(\chi (t_{0}))\int_{t_{0}}^{t}\zeta (\tau )\text{d}\tau )\\ \; & \; & \cdot a\exp(-a(t-t_{0}))-\exp(\chi (t_{0}))\\ \; & \; & \cdot \zeta (t)\exp(-a(t-t_{0})),\;\;\;\;t\in [t_{0},t_{1}]. \end{array}$$

When $t\geq t_{1}$, to obtain $\overset{˙}{\chi }=0,\chi =0$, ζ(t) must satisfy

(17) $$\left\{ \begin{array}{c} \zeta (t)=0,\;\;\;\;t<t_{0},t\geq t_{1},\\ \zeta (t_{0})=-\frac{\overset{˙}{\chi }(t_{0})+a\chi (t_{0})}{\exp(\chi (t_{0}))},\\ \int_{t_{0}}^{t_{1}}\zeta (\tau )\text{d}\tau =\frac{\chi (t_{0})}{\exp(\chi (t_{0}))},\\ \lim_{t\to t_{1}^{-}}\zeta (t)=0. \end{array}\right.$$

For an ILC system, it is only necessary to make

$$\sigma_{k}(t)={\overset{˙}{e}}_{k}(t)+ae_{k}(t)+r(t)$$

When $\sigma_{k}(t)=0$, only the appropriate r(t) must be chosen to make $e_{k}(t)=0,{\overset{˙}{e}}_{k}(t)=0$. Unfortunately, this method is only applicable to second-order systems. For the second-order system with only displacement shift, that is, for $\overset{˙}{\chi }(t_{0})=0$ in (17), Sun et al. [84], [85] introduced an attractor rectifying function into the sliding mode error, and achieved accurate tracking after a preset rectifying interval. Li et al. [86] improved the above method and solved the problem that the velocity also has an initial state shift. These methods [84], [85] gradually approach the corresponding differential equation solution through repeated iterations within a preset interval to finally achieve complete tracking.

**Figure 4:**
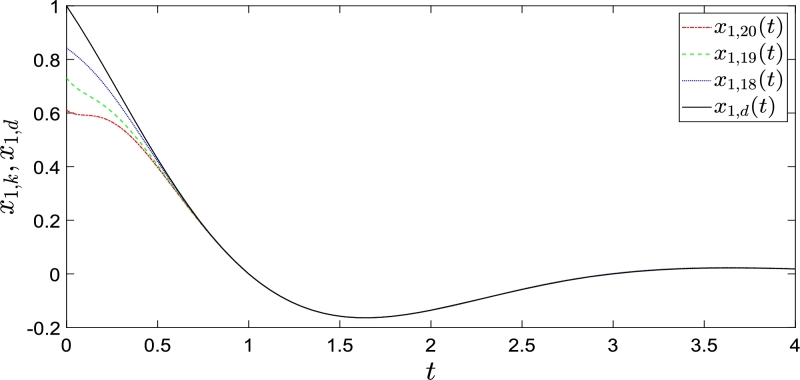
*x*_1,*k*_(*t*) and its desired trajectory *x*_1,*d*_(*t*).

**Figure 5:**
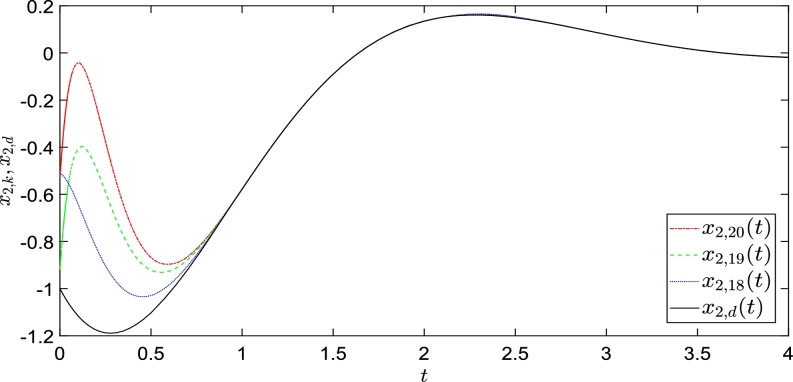
*x*_2,*k*_(*t*) and its desired state *x*_2,*d*_(*t*).

Consider the following second-order nonlinear continuous system:

$$\left\{ \begin{array}{ccc} {\overset{˙}{x}}_{1,k}(t)\; & =\; & x_{2,k}(t),\\ {\overset{˙}{x}}_{2,k}(t)\; & =\; & -(1+cost)x_{1,k}^{2}(t)cos(x_{1,k}(t))\\ \; & \; & +(1+t^{2})u_{k}(t)\\ y_{k}(t)\; & =\; & x_{1,k}(t), \end{array}\right.$$

where t∈[0,4]. The initial states of the system are $x_{1,k}(0)$ = 1−0.4rand, $x_{2,k}(0)$ = −0.5−0.5rand. The desired trajectories are $y_{d}(t)=x_{1,d}(t)=exp(-t)\cos(\frac{\pi }{2}t)$, $x_{2,d}(t)=-exp(-t)(\cos(\frac{\pi }{2}t)+\frac{\pi }{2}\sin(\frac{\pi }{2}t))$. We applied control law (11) and parameter update law (12), where the parameters were assigned according to the methods in [66]. The preset rectified initial state shift interval is [0,1). Twenty iterations are performed during the simulations and the results are presented in Figure 4, Figure 5. It can be seen that the system does not completely track the desired trajectory $x_{d}$ and desired state ${\overset{˙}{x}}_{d}$ in the preset interval [0,1). When t=1, $x_{1,k}$ and $x_{2,k}$ completely tracked desired trajectory $x_{d}$ and desired state ${\overset{˙}{x}}_{d}$, respectively.

For the following system with arbitrary initial state errors [87]

$${\overset{˙}{x}}_{k}(t)=\sum_{i=1}^{m}\theta^{i0}(t)\xi^{i0}(x_{k},t)+B(t)u_{k}(t)$$

we denote

$$x_{d,k}^{⁎}(t)=x_{d,k}(t)-\varphi_{h}(t)[x_{d,k}(0)-x_{d,k}(0)]$$

where $x_{d,k}(t)$ represents the reference trajectory, and

$$\varphi_{h}(t)=\left\{ \begin{array}{cc} {\left(1-\frac{t}{h}\right)}^{2},\; & t\in [0,h)\\ 0,\; & t\in [h,T] \end{array}\right.$$

The above definition shows that $x_{d,k}^{⁎}(t)$ represents the new reference trajectory after rectifying, which satisfies $x_{d,k}^{⁎}(0)=x_{k}(0)$, $x_{d,k}^{⁎}(t)=x_{d,k}(t)$, t∈[h,T]. Li et al. [87] utilized a rectified trajectory $x_{d,k}^{⁎}(t)$ and adaptive ILC to realize the complete convergence of the system. Jin [88] utilized the same idea to realize initial rectification for high-order systems. Sun et al. [89], [90], [91] used polynomials to construct the desired or error trajectories according to the initial state errors and completely tracked on the original trajectory after a preset time. In a sense, the methods [87], [88] rectify the desired trajectories, whereas methods [89], [90], [91], [92] reconstruct them. However, whether rectification or reconstruction of the reference trajectory or changing the error trajectory is required, the sliding mode error function (15) must be converted into

(18) $$\sigma_{k}^{⁎}(t)=c_{n}e_{k}^{(n),⁎}(t)+\cdots +c_{i}e_{k}^{(i),⁎}(t)+\cdots +c_{0}e_{k}^{⁎}(t)$$

The most obvious difference between (15) and (18) is that $e_{k}^{\left(i\right)}(0)\neq 0$ in (15) whereas $e_{k}^{(i),⁎}(0)=0$ in (18). Because $e_{k}^{(i),⁎}(0)=0$, that is, $\sigma_{k}^{⁎}(0)=0$, according to the Babalat lemma, one can easily get $\sigma_{k}^{⁎}(t)=0$, thereby ensuring $e_{k}^{(i),⁎}(t)=0$. If $e_{k}^{\left(i\right)}(0)\neq 0$, it is difficult to ensure that $e_{k}^{\left(i\right)}(t)=0$ even if $\sigma_{k}(t)=0$.

For nonlinear discrete systems with arbitrary initial states, Chi et al. [93] proposed an adaptive learning correction strategy to ensure that the system can achieve pointwise tracking. Compared with the correction strategy [94], the control ideas of the two did not differ significantly; however, the targeted systems were different. Chi et al. [93] focused on complex systems. When the initial state can be learned, the distributed fuzzy adaptive control law [95] realized the control of multiple agents. For a system with a non-repetitive process, although the control process does not satisfy repeatability or periodicity, and the system parameters change with time during the control process, if the bounds of the system parameters exist and are less than a certain constant, that is, the bounds of the system parameters are constants or they are invariant, the control law can be designed using the idea of ILC. When the desired trajectories were not repeatable, Li et al. [96], [97] adopted adaptive algorithms with initial correction functions and achieved satisfactory results.

#### 3.3.3 Initial state shifts compensation strategy

When the 2-D Roesser error model is used for system stability analysis, the system achieves asymptotic tracking when the initial state error is random within a certain bound [98].

Consider a 2-D FM system [94].

$$x_{k}(i+1,j+1)=A_{0}x_{k}(i,j)+A_{1}x_{k}(i+1,j)+A_{2}x_{k}(i,j+1)+Bu_{k}(i,j)$$

We define the error $e_{k}(i,j)=x_{d,k}(i,j)-x_{k}(i,j)$, where $x_{d,k}(i,j)$ is the iteratively changing desired trajectory. We obtain

$$\begin{array}{cc} \; & B^{-1}e_{k}(i+1,j+1)\;\\ =\; & B^{-1}(x_{d,k}(i+1,j+1)-x_{k}(i+1,j+1))\;\\ =\; & B^{-1}x_{d,k}(i+1,j+1)-B^{-1}A_{0}x_{k}(i,j)-B^{-1}A_{1}x_{k}(i+1,j)\;\\ \; & -B^{-1}A_{2}x_{k}(i,j+1)-u_{k}(i,j)\;\\ =\; & \theta \xi_{k}(i,j)-u_{k}(i,j)\; \end{array}$$

where $\theta =[B^{-1},-B^{-1}A_{0},-B^{-1}A_{1},-B^{-1}A_{2}]$, $\xi_{k}(i,j)={\left[x_{d,k}^{\text{T}}(i+1,j+1),x_{k}^{\text{T}}(i,j),x_{k}^{\text{T}}(i+1,j),x_{k}^{\text{T}}(i,j+1)\right]}^{\text{T}}$. The control and parameter update laws [94] are as follows:

(19) $$\begin{array}{c} u_{k}(i,j)={\overset{ˆ}{\theta }}_{k}\xi_{k}(i,j)\\ {\overset{ˆ}{\theta }}_{k}=proj({\overset{˜}{\overset{ˆ}{\theta }}}_{k})\\ {\overset{˜}{\overset{ˆ}{\theta }}}_{k}={\overset{ˆ}{\theta }}_{k}+\frac{Pe_{k-1}(i+1,j+1)\xi_{k-1}^{\text{T}}(i,j)}{\alpha +\xi_{k-1}^{T}(i,j)\xi_{k-1}(i,j)} \end{array}$$

where α>0, *P* is a positive definite diagonal matrix, and proj is the matrix saturation function. Utilizing the above adaptive iterative learning law, the control law ensures that $\lim_{k\to \infty }e_{k}(i,j)=0,i>0\&j>0$ when $2B^{-1}-P$ is a positive definite matrix. Therefore, for the 2−D system with an arbitrary initial state shift, adaptive iterative control is a better choice for achieving accurate tracking. The proof [94] shows that if the parameters are appropriately designed, the designed control law ensures that the error energy function decreases along the iterative axis regardless of the initial state error of the system, thereby ensuring that the system achieves accurate tracking.

To verify the tracking performance of the controller and the learning law (19) [94], we performed the following simulation. During the simulation, 500 iterations were performed and the control parameters were the same as those in [94]. The reference trajectory is presented below.

$$x_{dk}(i,j)=\left[\begin{array}{c} 2\sin(0.1t+0.77j),\;\;\;0\leq i\leq 20\\ 3\cos(0.29t+0.5j),\;\;\;0\leq j\leq 20 \end{array}\right]$$

**Figure 6:**
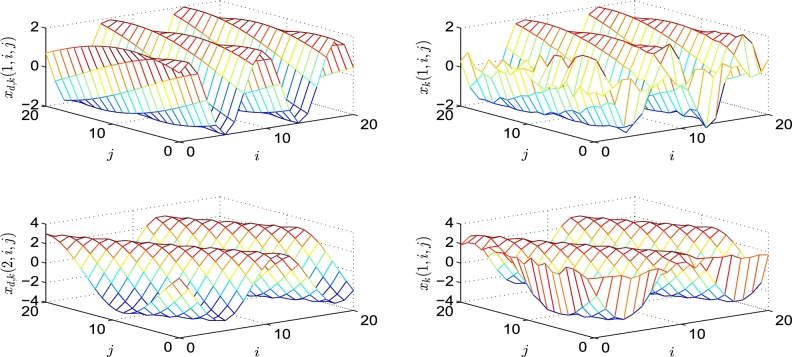
The 500th tracking trajectories *x*_*k*_(1:2,*i*,*j*) and their reference trajectories *x*_*dk*_(1:2,*i*,*j*).

**Figure 7:**
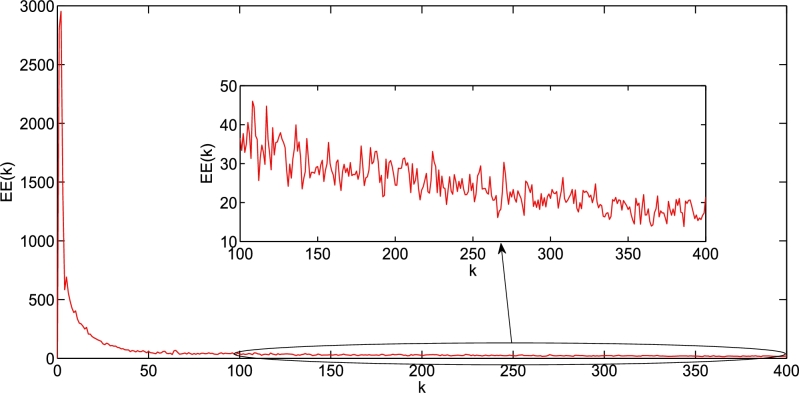
*EE*(*k*).

The initial states of $x_{k}(i,j)$ are as follows.

$$x_{k}(i,1)=\begin{array}{c} {}[1⁎rand;1.5+1⁎rand],\;\;\;0\leq i\leq 20 \end{array}\;x_{k}(1,j)=\begin{array}{c} {}[1⁎rand;1.5+1⁎rand],\;\;\;0\leq j\leq 20 \end{array}$$

where k denotes the number of iterations. The simulation results are shown in Figure 6, Figure 7. In Fig. 6, the left and right sides show the reference trajectories $x_{dk}(i,j)$ and the 500th tracking trajectories $x_{k}(i,j)$, respectively.

Because of its three-dimensional shape, which is unsuitable for comparison, we present a better illustration through the error in Fig. 7. Here, the tracking error EE(k) is defined as follows:

$$\begin{array}{c} EE(k)=\sum_{i=0}^{20}\sum_{j=0}^{20}{\left[e_{k}{\left(1:2,i,j\right)}^{\text{T}}e_{k}(1:2,i,j)\right]}^{\frac{1}{2}} \end{array}$$

Moreover, the inset in Fig. 7 clearly shows the asymptotically converging trend of the error EE(k) along the iteration axis.

## 4 Optimal controllers and rectifying algorithms

### 4.1 Optimal controllers

To ensure the control effect, optimal ILC has received increasing attention [99]. The optimization method generally refers to the method of reaching the optimal value of the objective function after satisfying the constraints. In ILC, optimization methods can be divided into two categories: model-based optimization and data-driven optimization methods [100], [101], [102].

For systems with models, Amann et al. [103] proposed a non-causal optimal ILC law by optimizing the following objective function based on the gradient method, which optimized the vector norm, and the algorithm reached the geometric level of the convergence speed.

$$J_{k+1}(u_{k+1})={\left\|e_{k+1}\right\|}_{Y}^{2}+{\left\|u_{k+1}-u_{k}\right\|}_{U}^{2}$$

where $e_{k}$ and $u_{k}$ represent the tracking error and the control signal at the kth iteration, respectively. Lim et al. [104] proposed a Pareto learning control framework that integrated multiple indicators into a design framework to improve the multiple performance indicators of the system. Axelsson et al. [105] modified the objective function in the optimization problem, extended the norm-optimal ILC algorithm for linear systems, and applied the Kalman filter to linear time-invariant systems. Cao et al. [106], [107] combined ILC with a robust Kalman filter for discrete repetitive processes to determine the system state.

Data-driven control (DDC) is a theory and method for controller design that directly uses online or offline I/O data of the controlled system or knowledge from data processing without using explicit or implicit mathematical model information [100]. To date, there have been several types of DDC methods [108], [109], [110], [111]. Compared with traditional optimal ILC, the controller design and analysis of the DDC method relies primarily on the online or offline I/O data of the system. Notably, the DDC method does not exclude known model information.

### 4.2 Initial rectifying algorithms

Owing to the strong anti-interference ability of the optimal control method, the strategy of applying the optimal control method and implementing the initial state shifts rectifying is rare. Liu et al. [112] presented a terminal ILC method based on a neural network for a class of uncertain nonlinear non-affine systems. A neural network was used to approximate the initial state and effectively suppress the interference of the initial state shifts, resulting in a better tracking effect. The method presented by Liu et al. [113] is similar to that described above but for different systems. For the linear discrete system (3), the following equation holds:

$$\begin{array}{cc} y_{k}(N)\; & =CA^{N}x_{k}(0)+C\sum_{t=0}^{N-1}A^{N-t-1}Bu_{k}\\ \; & =f(x_{k}(0))+B^{⁎}u_{k} \end{array}$$

where $f(x_{k}(0))=CA^{N}x_{k}(0)$, $B^{⁎}=C\sum_{t=0}^{N-1}A^{N-t-1}B$. Simultaneously, the neural network is constructed to approximate $f(x_{k}(0))$, that is, $f(x_{k}(0))=W^{\text{T}}\Phi (x_{k}(0))$. If $\Theta =[W^{\text{T}},B^{⁎}]$, $\Psi_{k}={\left[\Phi {\left(x_{k}(0)\right)}^{\text{T}},u_{k}^{\text{T}}\right]}^{\text{T}}$, the neural network system has an approximate terminal output $y_{k}^{NN}(N)=\Theta \Psi_{k}$. High-precision tracking was achieved by the adaptive learning of Θ.

**Figure 8:**
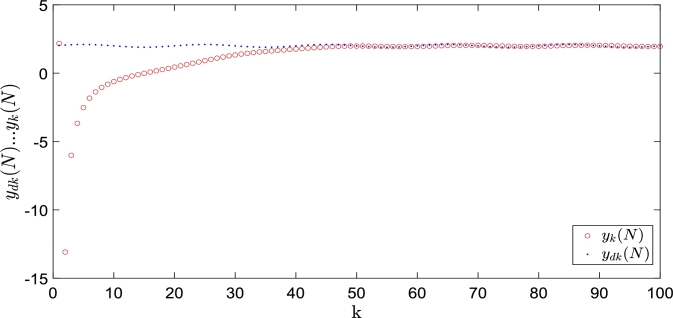
*y*_*k*_(*N*) and its desired trajectory *y*_*dk*_(*N*).

**Figure 9:**
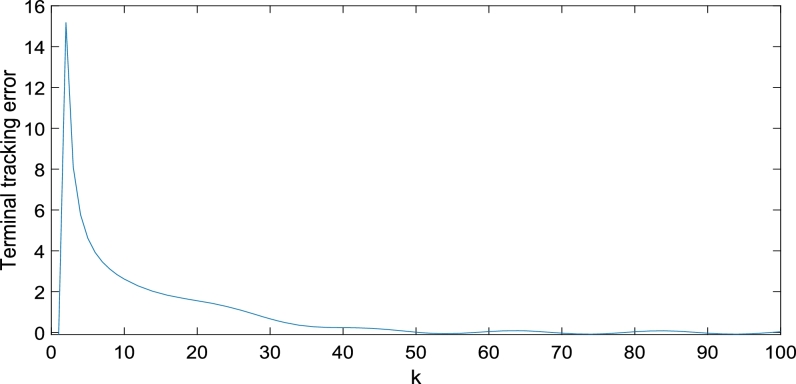
Terminal tracking error.

The control and learning laws are applied, where the parameters and target trajectory are the same as those in [112]. The simulation results presented in Figure 8, Figure 9, indicate that the tracking effect is satisfactory. This method avoids the uncertainty caused by the initial state errors and solves the inconsistency of the initial state of the discrete system; thus, it is robust.

For model-free repeatable systems with stochastic initial conditions,

$$\begin{array}{cc} y_{k}(t+1)\; & =f(y_{k}(t),y_{k}(t-1),\cdots ,y_{k}(t-n_{y}),u_{k}(t),u_{k}(t-1),\cdots ,u_{k}(t-n_{u})) \end{array}$$

According to the method proposed by Chi et al. [114], the following formula can be obtained.

$$\begin{array}{cc} y_{k}(N)\; & =g_{N-1}(y_{k}(0),u_{k}(0),u_{k}(1),\cdots ,u_{k}(N-1)) \end{array}$$

For a function $g_{N-1}(\cdot )$ that satisfies the Lipschitz condition, and denoting ${\text{U}}_{k}={\left[u_{k}(0),u_{k}(1),\cdots ,u_{k}(N-1)\right]}^{T}$, $\Delta y_{k}(t)=y_{k}(t)$ $-y_{k-1}(t)$, $\Delta {\text{U}}_{k}={\text{U}}_{k}-{\text{U}}_{k-1}$, then

(20) $$\begin{array}{c} \Delta y_{k}(N)=\theta_{1,k}+\theta_{2,k}^{\text{T}}\Delta {\text{U}}_{k} \end{array}$$

Here, $\theta_{1,k}=\frac{\partial g_{N-1}^{⁎}}{\partial y_{k}(0)}\Delta y_{k}(0)$ is the optimal partial derivative, which represents the impact of the initial state errors on the terminal output; $\theta_{2,k}=[\frac{\partial g_{N-1}^{⁎}}{\partial u_{k}(0)}$, $\frac{\partial g_{N-1}^{⁎}}{\partial u_{k}(1)}$, ⋯, ${\frac{\partial g_{N-1}^{⁎}}{\partial u_{k}(N-1)}]}^{\text{T}}$ is the unknown gradient parameter vector. Equation (20) can be rewritten as follows:

$$\begin{array}{c} y_{k}(N)=y_{k-1}(N)+\theta_{1,k}+\theta_{2,k}^{\text{T}}\Delta {\text{U}}_{k} \end{array}$$

Then, in the learning law, the $\theta_{1,k}$ that contains the unknown initial state shifts is iteratively estimated, and the bound of the tracking error is obtained. Chi et al. [115] used a high-order internal model adaptive ILC to overcome initial state shifts. In a sense, the methods [112], [113], [114], [115] only compensate for the initial state shifts and rectification is not achieved. Yan et al. [116] used suboptimal control to solve the ILC problem of nonlinear uncertain systems with random initial state shifts. This method rectified the initial state errors and ensured that the system completely track the desired trajectory within a defined interval.

## 5 Conclusion

This paper introduces three types of iterative learning controllers: PID-type, adaptive, and optimal. To address the interference caused by arbitrary initial state shifts when applying these three controllers, considerable amount of technical analysis has been conducted on the relevant initial state shifts rectifying measures. Thus far, if there are random initial state errors in each iteration, accurate tracking can be achieved in a specified interval using the adaptive iterative learning controller and attractor method. However, for PID-type controllers and optimal ones, there are few methods to achieve accurate tracking within a defined interval when there are arbitrary initial state shifts. Application of these two control methods can achieve asymptotic tracking. In particular, for a PID-type controller, it is more practical to rectify arbitrary initial state shifts under the contraction mapping framework. However, for discrete systems with random initial state errors, the inability to use integration methods increases the difficulty of rectifying the initial state errors. In addition, in the field of ILC, there are still many topics related to initial state shifts, as listed below.

1. The application of matrix theory or other analytical methods to analyze the stability of systems with random initial state errors, especially the stability analysis of discrete time-delay systems and nonlinear systems, is worth investigating.

2. The application of ILC to non-repetitive processes with the help of iterative learning initial rectifying technology to achieve rapid convergence of the system. Each control method has its advantages and disadvantages. If different control methods can be combined and practically implemented, their advantages will complement each other.

Thus many open topics regarding the initial rectifying of ILC remain to be studied, and researchers need to develop and perfect this technique.

## CRediT authorship contribution statement

All authors listed have significantly contributed to the development and the writing of this article.

## CRediT authorship contribution statement

**Dongjie Chen:** Conceptualization, Software, Validation, Writing – original draft, Writing – review & editing. **Tiantian Lu:** Software, Writing – review & editing. **Guojun Li:** Conceptualization, Validation, Writing – review & editing.

## Declaration of Competing Interest

The authors declare that they have no known competing financial interests or personal relationships that could have appeared to influence the work reported in this paper.

## Data Availability

Data included in article/supp. material/referenced in article.
